# An original donor-dependent spheroid system for the prediction of idiosyncratic drug-induced liver injury risk

**DOI:** 10.1007/s44164-023-00057-w

**Published:** 2023-08-15

**Authors:** Sara Cherradi, Nicolas Taulet, Hong Tuan Duong

**Affiliations:** PredictCan Biotechnologies SAS, Biopôle Euromédecine, Grabels, France

**Keywords:** Educated spheroid, Drug-induced liver injury, Hepatotoxicity, Preclinical model, Non-genetic host factor risk

## Abstract

One major drawback of preclinical models to test drug-induced liver injury (DILI) is their inability to predict the interindividual difference of DILI effect in a population. Consequently, a high number of molecules that passed preclinical phases, fail clinical trials, and many FDA-approved drugs were removed from the market due to idiosyncratic DILI. We use a proprietary-depleted human serum-based cell educating technology to generate donor-dependent spheroids with distinct morphology and functionality. We demonstrate that educated spheroids could capture the large variations in susceptibility to drug-induced liver injury between donors. We show that the model could predict clinical apparent DILI risk with a high specificity and sensitivity. We provide evidence that the model could address non-genetic factor-associated DILI risk and severity such as age or sex. Our study supports the benefit of using donor-dependent educated spheroids for hepatotoxicity evaluation in preclinical phase or in an exploratory study clinical trial phase 2 to provide a robust safety profile to a drug.

## Introduction

The lack of sufficient compliance between preclinical models, including non-animal and animal models, and human physiology is a major cause of poor efficacy or of high toxicity of a drug when entering clinical trials [1, 2]. It is well accepted that people’s susceptibility in drug responsiveness and drug-induced liver injury (DILI) is the main challenge in drug development and precision medicine [3, 4]. Nevertheless, it is currently impossible to test the interindividual variability of drug-mediated cellular responses before initiating clinical trials because of the lack of models that mimic that interindividual difference in a population [5]. Therefore, the generation of in vitro systems capable of mimicking cell functionality of human livers of a representative population to analyze drug-induced hepatotoxicity is necessary for the determination of safe medication dose ranges.

Because in normal physiological as well as in pathological conditions liver cells functions are not exclusively modulated by the intra-organ microenvironment but also by the inter-organ communication through plethora of released compounds including soluble factors, exosomes, and gut microbiota-derived metabolites and products that are found in the bloodstream [6–13], we developed a method that utilize depleted serum from each person to educate hepatic cell lines cultured as spheroids, to phenotypically mimic the interindividual difference in drug responsiveness.

We show that donor-dependent educated spheroids can predict clinical apparent DILI risk with a high specificity and sensitivity. Importantly, we demonstrate that our system could be used to assess non-genetic host factors such as age or sex that are linked to DILI risk and severity. To our knowledge, this is the first easy to set up human-derived model that better represents the variation of the human population making it a perfect tool to de-risk DILI for new compounds in development in pre-phase 1 or to provide a more robust safety profile to the drug in an exploratory study clinical trial phase 2.

## Material and methods

### Reagents, depleted serum, and cells

Blood samples are provided by the Etablissement Français du Sang (EFS) Hauts de France–Normandie. Depleted human serum was obtained after a filtration step through a 0.45-μm mesh filter. The study was approved by the “Direction Générale de la recherche et de l’innovation” (CODECOH, n°DC-2021-4779). This project does not involve the human person according to the legislation (article L1121-1 du code de la santé publique). Albuterol, flavoxate, etoposide, β-estradiol, nizatidine, azathioprine, oxaliplatin, bosentan, sorafenib, cabozantinib, lenvatinib, rifampicin, and stavudine were purchased from CliniSciences (Nanterre, France). Hepatocyte (HepG2) and hepatic stellate cell lines (TWNT-1) were from ATCC (Molsheim, France) and Glow Biologics (Tarrytown, NY, USA), respectively. All cell culture reagents were provided by StemCell (Saint Égrève, France). Hepatocytes and hepatic stellate cells were conditioned for a minimum of 2 weeks in MammoCult® basal medium (StemCell) before use, to sensitize them to the cell educating technology. Absence of mycoplasma contamination was verified using MycoAlert® Mycoplasma Detection Kit from Lonza (Saint-Beauzire, France).

### Generation of educated spheroids and treatments

Educated spheroids were generated from a co-culture of HepG2 and TWNT-1 cell lines in MammoCult® basal medium supplemented with depleted human serum for 3 days in 384 wells ultra-low attachment plates (Dutscher SAS, Bernolsheim, France). A dose-dependent treatment ranging from 0.01× to 100× *C*_max_ for each compound was performed for up to 96 h on educated spheroids. Cell viability was measured using CellTiterGlo (Promega, Charbonnières-les-Bains, France) according to the manufacturer’s instructions.

### Cytochrome P450 activity and total collagen type I quantification

CYP3A4 activity and total collagen deposition were assessed using P450-Glo™ CYP3A4 Assay (Promega, Charbonnières-les-Bains, France) and Total Collagen Assay Kit (Perchlorate-Free) (ab222942, Abcam, Paris, France) according to the manufacturer’s instructions, respectively.

### Immunofluorescence

Donor-dependent educated spheroids were cultured for 3 days in 96 wells ultra-low attachment plates (Dutscher, Bernolsheim, France). Spheroids were fixed with PBS-10% PFA (Fisher Scientific, Illkirch, France) for 30 min, permeabilized with PBS-0.5% Triton X-100 (Sigma-Aldrich, Saint-Quentin-Fallavier, France) for 2 h, and incubated in blocking buffer (0.2% Triton X-100, Bovine Serum Albumin (Euromedex, Souffelweyersheim, France) in PBS) for 2 h at room temperature. Primary antibodies, including FITC-α-tubulin (F2168, Sigma-Aldrich), type 1 collagen (COL1A1, #72026, Cell Signaling, Ozyme, Saint-Cyr-L’École, France), Fibronectin (FN1, #26836, Cell signaling), FITC-α-smooth muscle Actin (ab8211, Abcam), ZO-1 (#61-7300, Life Technologies SAS, Courtaboeuf Cedex, France), and MRP2 (#4446, Cell Signaling) antibodies, were diluted in blocking buffer and incubated overnight at 4 °C. After washes with blocking buffer, the secondary antibody (anti-rabbit Alexa Fluor 555, #4413, Cell Signaling) was added for 3 h at room temperature followed by nuclei staining with DAPI (#4083, Cell Signaling). Spheroids were transferred into μ-Slide 8 Well (Ibidi, CliniSciences, Nanterre, France), and images were acquired on a Dragonfly spinning disk confocal microscope (Andor, Oxford Instruments, High Wycombe, UK) equipped with EMCCD iXon888 Life Andor camera (Objective 20X/0.75 NA) and controlled by Fusion software (Andor). Fluorescence intensity was quantified using ImageJ software. Values were obtained from z-stack projections (sum slices) and correspond to the median values of the pixels in the images after background subtraction. 3D views were performed with Imaris software (Bitplane).

### RNA sequencing

RNAseq experiments were performed by Acobiom (Grabels, France). RNA extraction was performed using miRNeasy kit (Qiagen, Courtaboeuf, France), with on-column DNase digestion according to manufacturer’s instructions. Briefly, educated spheroids were homogenized in 700 μl QIAzol® Lysis Reagent in a 2-ml SafeLock microcentrifuge tube. One 2-mm stainless steel bead was added to each sample and they were disrupted by mechanically using TissueLyzer (Qiagen) 2 × 2 min at 20 Hz. Samples were then incubated 5 min at room temperature. One hundred forty microliters chloroform was added to the homogenate. Tubes were shaked vigorously for 15 s, and they were placed back onto the benchtop for another 3 min. Lysates were centrifuged at 12,000 x *g* for 15 min at 4 °C in a microcentrifuge. Upper aqueous phases were carefully transferred to clean 2-ml microcentrifuge tubes. RNA was eluted in water and immediately stored at − 80 °C until use. The full procedure was performed using QIAcube automated workstation (QIAcube–QIAGEN) to optimize reproducibility of RNA extraction. RNA integrity was assessed using Agilent 2200 TapeStation with RNA ScreenTapes. RINe (RNA Integrity Number equivalent) scores were > 7.7 for all samples. RNA-seq libraries were prepared following the protocol TruSeq Stranded Total RNA and validated on labchip GX platform. Human GRCh38.p13 genome was used as a reference. RNA-Seq data were mapped and annotated using Ensembl database release 108 (https://www.ensembl.org).

### Graphs and statistics

Plots and statistics were generated using GraphPad Prism v9 (Dotmatics, San Diego, CA); otherwise, Excel (Microsoft Office 364).

## Results

**Fig. 1:**
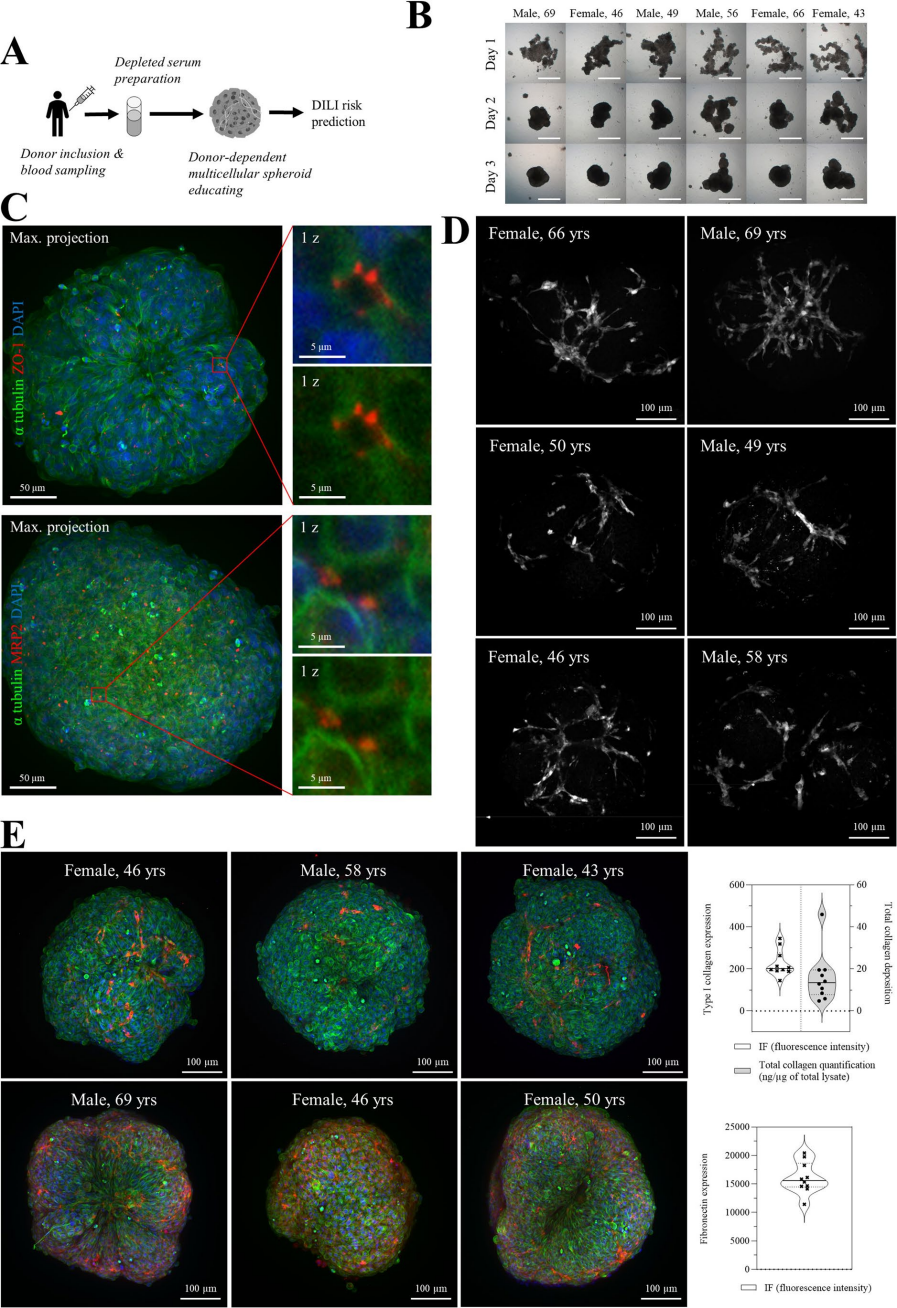
Donor-dependent educated spheroids display a distinct phenotype and ECM production. **A** Workflow of cell line-based spheroids educating. **B** Spheroids were educated for 3 days with donor’s sera. Pictures show the phenotypes at days 1, 2, and 3. Scale bar: 250 μm. **C** Formation of bile canalicular structure. Educated spheroids were generated with the depleted serum of a 41-year-old female and then stained for ZO-1 and MRP2. **D** Activation of hepatic stellate cells. Educated spheroids from 6 different donors were stained for α-SMA after 3 days of culture. Scale bar: 100 μm. **E** Educated spheroids from different donors were stained for type 1 collagen, fibronectin, and α-tubulin after 3 days of culture. Violin plots (upper right) show a quantification of type 1 collagen protein deposition by immunofluorescence and by colorimetric assay for 10 different donors. Violin plot (lower right) shows the quantification of fibronectin deposition for 10 different donors. Each dot corresponds to one donor. Solid line is the median. Dotted thin black lines show quartiles

We used depleted serum prepared from blood sampling of healthy donors to educate spheroids containing human hepatic and human stellate cell lines (HepG2 and TWNT-1) (Fig. 1A). By adding depleted human serum to the cell culture medium, we observed that the rate of autonomous spheroid formation varies between donors, and their shapes are different after 3 days of culture (Fig. 1B). Confocal microscopy analysis revealed that the spheroids are positive for ZO-1, a tight junction protein [14], and MRP2, an ATP-binding cassette transporter that has an important role in the detoxification and chemoprotection [15], suggesting that educated spheroids contain functional bile canalicular structures (Fig. 1C). We showed also that the level of activation of hepatic stellate cells is donor-dependent (Fig. 1D), and consequently, we observed that the amount of spontaneous deposition of extracellular matrix (ECM), such as type I collagen and fibronectin, varies also between donors (Fig. 1E).

**Fig. 2:**
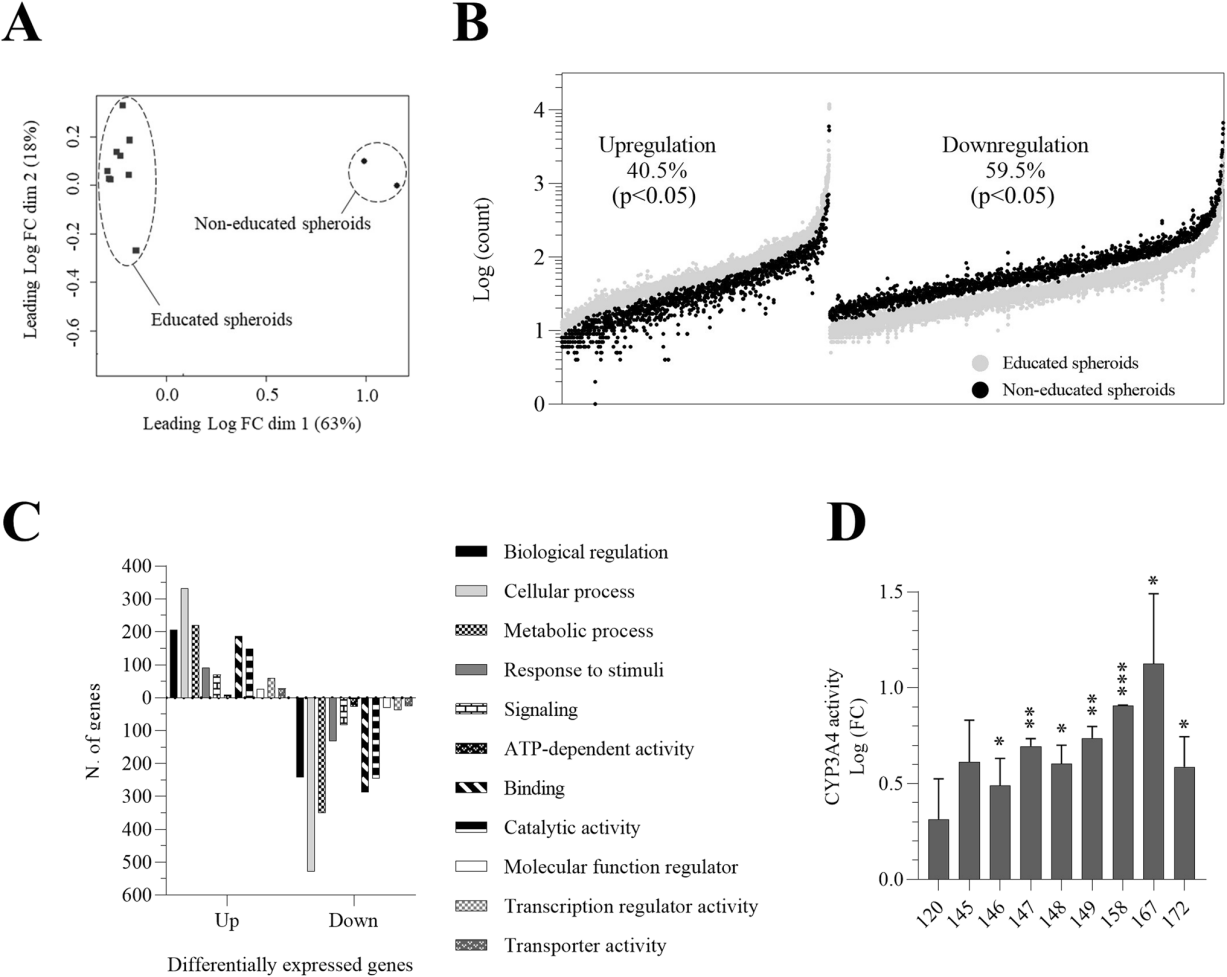
Alteration of the transcriptomic profile and upregulation of the basal CYP3A4 activity in educated spheroids. **A** Principal component analysis separates transcripts from educated and non-educated spheroids. Educated spheroids from 10 different donors and non-educated spheroids were sequenced after 3 days of culture. **B** Analysis of differentially expressed genes (DEGs). 1460 DEGs were found after the educating step. Fisher’s *t*-test **C** Gene Ontology (GO) analysis of DEGs using PANTHER classification system. Graph shows the number of differentially expressed genes compared to non-educated condition with *p* < 0.05. **D** Increased CYP3A4 basal activity in educated spheroids as compared to non-educated spheroids. CYP3A4 activity was measured after 3 days of culture. Shown are the results from 9 donors. Results are expressed as mean ± s.e.m. of Log fold change to non-educated spheroids. *p < 0.05, **p < 0.01, ***p < 0.001, Fisher’s *t*-test

To further characterize donor-dependent educated spheroids, their molecular signatures were assessed by RNAseq. Principal component analysis (PCA) showed a clear separation of educated spheroids from non-educated spheroids (Fig. 2A). Analysis of differentially expressed genes (DEGs) indicates that the expression of 1460 genes differs between educated and non-educated spheroids. Among those 1460 DEGs, we found that 591 genes (40.5%) were upregulated while 869 DEGs (59.5%) were downregulated after the educating step (Fig. 2B). Gene Ontology analysis showed that these differentially expressed genes are assigned to biological regulation, cellular, metabolic, signaling, ATP-dependent, response to stimuli, and binding processes, as well as to catalytic, regulatory, and transport activities (Fig. 2C). Interestingly, we found also that educated spheroids showed an increase in CYP3A4 basal activity by 2 to 19 times as compared to non-educated spheroids (Fig. 2D).

**Fig. 3:**
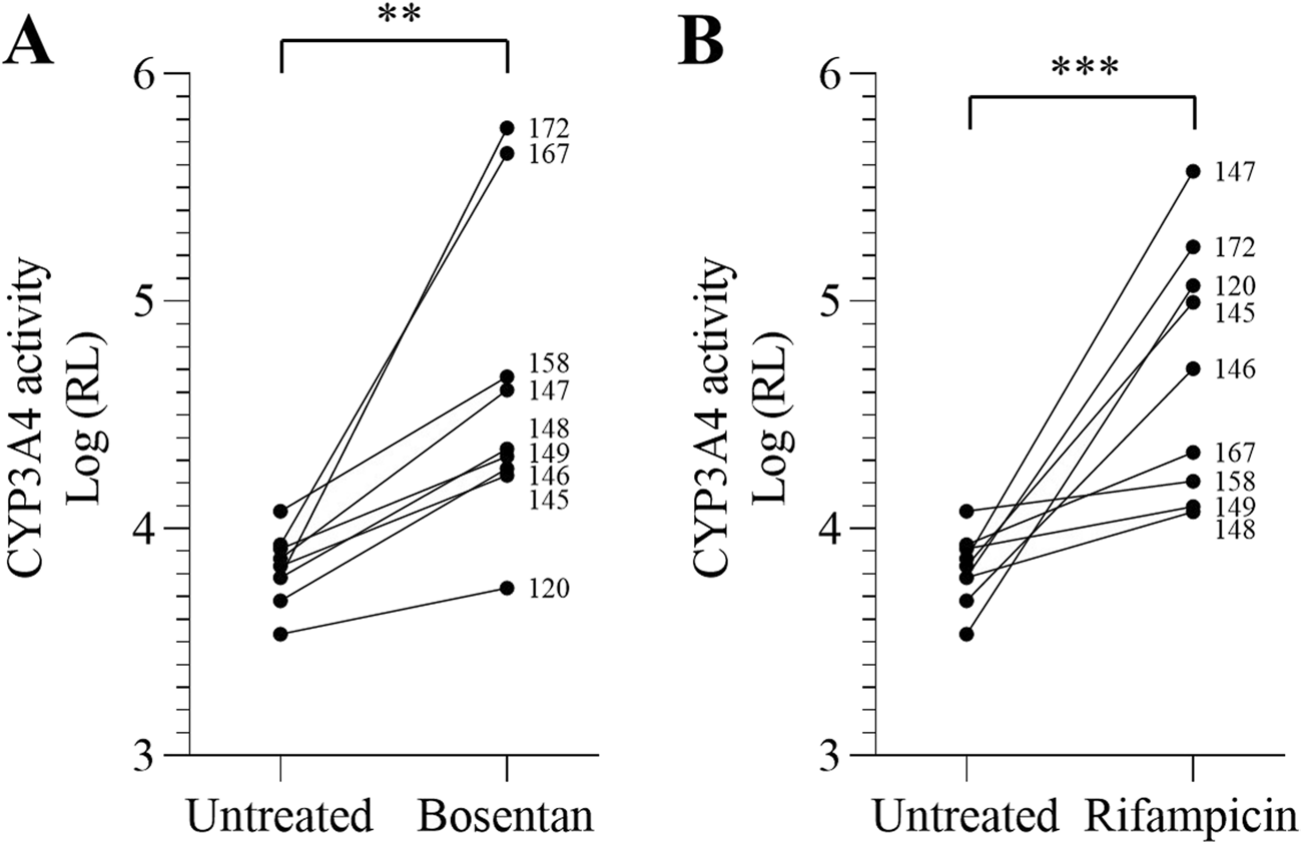
Induction of drug metabolizing capacity in educated spheroids. CYP3A4 activity was measured in educated spheroids from 9 different donors after 4 days of exposure to bosentan (**A**) or to rifampicin (**B**). Results are shown as Log relative luminescence. Educated spheroids significantly increased CYP3A4 activity in response to bosentan and rifampicin. **p < 0.01, ***p < 0.001, Mann-Whitney *t*-test

The induction of the activity of CYP3A4 that metabolizes about half of all drugs on the market [16] was assessed after treatment for 4 days with bosentan and rifampicin. As expected, we found an enhanced metabolizing CYP activity in a donor-dependent manner, ranging from 1.5 to 80 times upon bosentan treatment (Fig. 3A) and from 1.5 to 55 times upon rifampicin treatment (Fig. 3B). Our data indicate that donor-dependent educated spheroids may be valuable experimental tools for predicting drug metabolism and thus drug-induced liver injury.

**Fig. 4:**
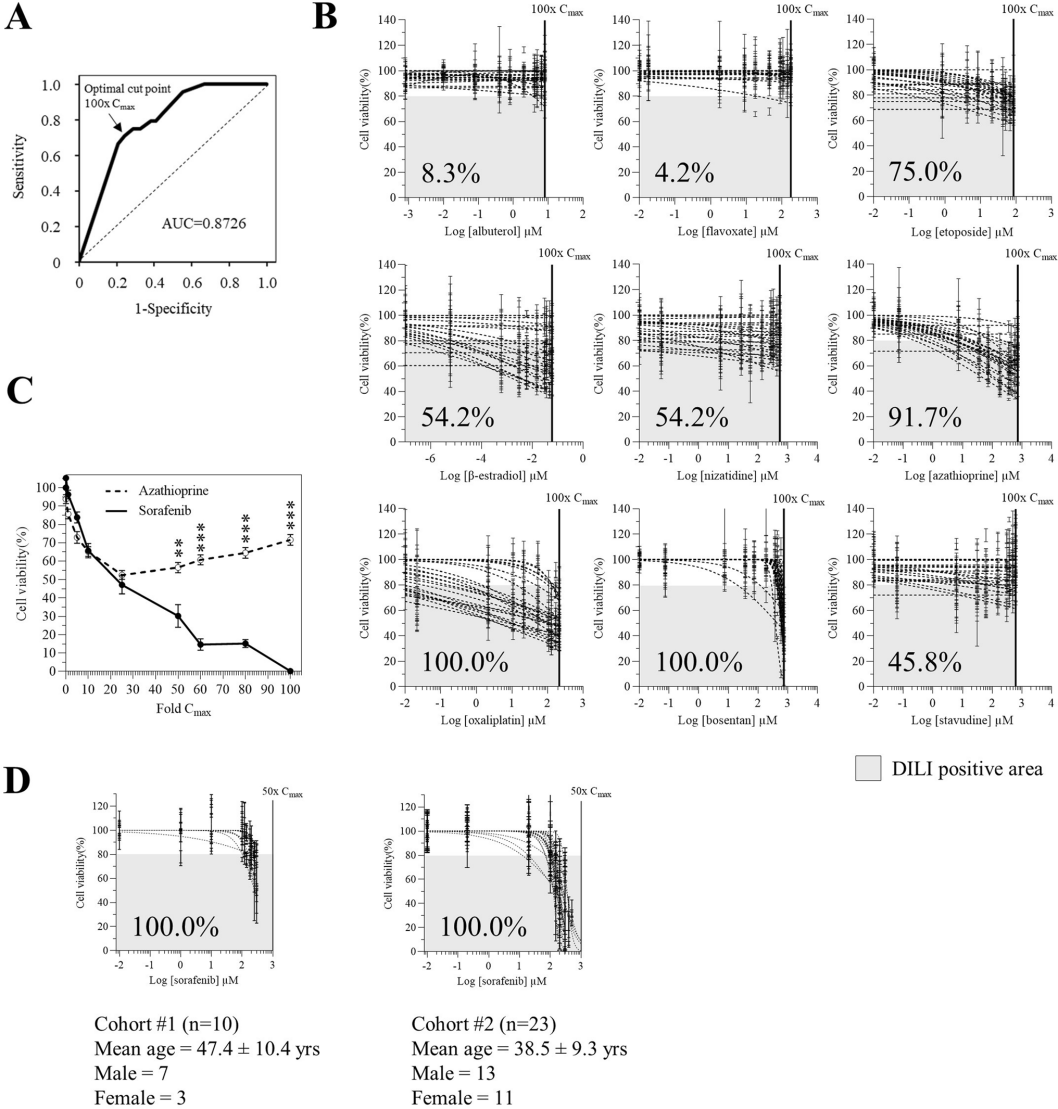
Prediction of DILI risk by educated spheroids. **A** Receiver-operating curve (ROC) analysis of MOS_20_ as predictor of clinical DILI. ROC curve was generated from MOS_20_ of each donor of the independent groups, and the optimal cut point was determined. **B** Inhibitory dose-response curve fit with constrains (top = 100; bottom = 0) for each drug. DILI positive area is determined by the range [20% reduction of cell viability–100× *C*_max_]. The percentage on the graph indicates the proportion of donors within a cohort of 24 donors, showing a DILI positive mark. Results are shown as percentage of cell viability of at least a triplicate. **C** Ability of educated spheroids to detect iDILI drug in dose-independent manner. For each drug, educated spheroids from 24 donors were used. Treatment duration was 96 h, and the concentrations range from 0.01× to 100× *C*_max_. Results are shown as percentage of cell viability of at least a triplicate. **p < 0.01, ***p < 0.001, Mann-Whitney *t*-test. **D** Reliability of educated spheroids to predict DILI risk. The experiment was performed on 2 independent cohorts. The concentrations used range from 0.01× to 50× *C*_max_

To test whether donor-dependent educated spheroids could estimate the actual DILI in a population, we experimentally generated treatment groups of 24 randomly selected individuals (from *n* = 109 donors) and performed a DILI risk prediction. The number of donors included in each group was determined based on the study published by Fermini and colleagues, where the authors reported that a sample size of 24 is sufficient to have 92% of chance to detect an event with 10% incidence [17]. The age, the sex, and the ABO blood type of the donors are reported in Table 1. Educated spheroids were treated with a panel of drugs with clinical apparent DILI. These drugs are known as difficult-to-detect DILI compounds by current preclinical models. The concentrations of the drugs used range from 0.01× *C*_max_ to 100× *C*_max_. DILI risk is determined using the numerical margin of safety (MOS) [18–21] calculated with the drug concentration that induced at least 20% of cell death. ROC curve analysis showed that MOS_20_ can discriminate DILI-positive drugs from DILI-negative drugs with an optimal cut point at 100× *C*_max_ and an area under the curve (AUC) of 0.8726 (Fig. 4A). For each drug and for each donor included in the study, we generated an inhibitory dose-response curve fit with constrains (top = 100; bottom = 0) and calculated the LogIC50 and HillSlope values (Table 2). The DILI risk is estimated by using the toxicity score (TS) that is calculated with the formula reported in Table 2. A drug is considered as at clinical DILI risk if at least 10% of individuals within the cohort are categorized as DILI positive (based on the TS) (Table 2). As expected, we found a variation in the susceptibility to drug-induced liver injury between donors. We observed that in the cohorts that were treated with albuterol or with flavoxate, only 8.3% and 4.2% of donors were DILI positive confirming that these drugs have no clinical DILI concerns (Fig. 4B). In contrast, in cohorts that were treated with etoposide, β-estradiol, nizatidine, azathioprine, oxaliplatin, bosentan, and stavudine, 75%, 54.2%, 54.2%, 91.7%, 100%, 100%, and 45.8% of donors, were DILI positive, respectively, confirming that these drugs are clinically at high risk for DILI development (Fig. 4B; Table 3).

**Table 1:** Donor’s characteristics

Donor #	Sex	Age	ABO blood type	Used in figure	Donor #	Sex	Age	ABO blood type	Used in figure
11	M	24	O	4D	89	M	25	O	4A, 4B, 4C, 5, 6
12	M	44	O	4D	90	M	27	O	4A, 4B, 4C, 5, 6
13	F	67	A	4D	91	F	24	O	4A, 4B, 4C, 5, 6
14	M	46	A	4D	92	M	51	B	4A, 4B, 4C, 5, 6
15	F	33	A	4D	93	F	23	O	4A, 4B, 4C, 5, 6
16	M	57	O	4D	94	F	38	O	4A, 4B, 4C, 5, 6
17	M	38	O	4D	95	M	59	O	4A, 4B, 4C, 5, 6
18	F	52	O	4D	96	M	42	O	4A, 4B, 4C, 5, 6
19	M	61	B	4D, 6	97	F	54	O	4A, 4B, 4C, 5, 6
20	M	52	A	4D	98	M	37	A	4A, 4B, 4C, 5, 6
21	M	69	B	1B, 1D, 2	99	F	27	O	4A, 4B, 4C, 5, 6
22	F	47	O	4A, 4B, 5, 6	100	F	23	O	4A, 4B, 4C, 5, 6
23	F	46	A	1B, 1D, 1E, 2	101	M	62	A	4A, 4B, 4C, 5, 6
24	F	43	A	1E, 2	102	F	30	O	4A, 4B, 4C, 5, 6
25	M	49	O	1B, 1D, 2	103	F	50	O	4A, 4B, 5, 6
26	M	56	A	1B, 1E, 2	104	M	26	B	4A, 4B, 5, 6
27	F	65	O	4A, 4B, 5, 6	105	F	41	A	4A, 4B, 5, 6
28	M	63	AB	4A, 4B, 5, 6	106	F	20	O	4A, 4B, 5, 6
29	F	50	A	4A, 4B, 5, 6	107	M	20	B	4A, 4B, 5, 6
30	F	66	O	1B, 1D, 1E, 2	108	F	30	A	4A, 4B, 5, 6
31	F	50	O	1D, 2	109	M	24	A	4A, 4B, 5, 6
32	F	55	A	4A, 4B, 5, 6	110	F	36	O	4A, 4B, 5, 6
33	F	43	B	1B, 1E, 2	111	F	50	A	4A, 4B, 5, 6
34	M	58	A	1D, 1E, 2	112	M	63	O	4A, 4B, 5, 6
35	F	41	O	4A, 4B, 5, 6	113	F	22	B	4A, 4B, 5, 6
36	F	51	A	4A, 4B, 5, 6	114	M	18	B	4A, 4B, 5, 6
37	M	51	A	4A, 4B, 5, 6	115	M	30	O	4A, 4B, 5, 6
38	F	36	O	1E, 4A, 4B, 5, 6	118	F	21	A	6
39	F	36	A	4A, 4B, 5, 6	119	F	23	O	6
39	F	36	A	6	120	F	20	A	2D, 3A, 3B, 6
40	M	50	O	4A, 4B, 5, 6	121	F	45	A	4A, 4B, 5, 6
41	F	38	O	4A, 4B, 5, 6	122	F	65	A	4A, 4B, 5, 6
42	M	50	A	4A, 4B, 5, 6	123	F	50	O	4A, 4B, 5, 6
43	F	70	A	4A, 4B, 5, 6	124	F	64	A	4A, 4B, 5, 6
44	F	34	A	4A, 4B, 5, 6	125	M	60	AB	4A, 4B, 5, 6
45	M	38	A	4A, 4B, 5, 6	126	F	49	AB	4A, 4B, 5, 6
46	F	46	A	4A, 4B, 5, 6	127	M	63	AB	4A, 4B, 5, 6
47	F	46	O	4A, 4B, 5, 6	128	M	58	AB	4A, 4B, 5, 6
48	F	34	O	4A, 4B, 5, 6	129	F	40	O	4A, 4B, 5, 6
49	F	60	A	4A, 4B, 5, 6	130	F	36	O	4A, 4B, 5, 6
50	F	32	O	4A, 4B, 5, 6	131	F	44	AB	4A, 4B, 5, 6
51	F	33	A	4A, 4B, 5, 6	132	M	47	AB	4A, 4B, 5, 6
52	F	56	A	4A, 4B, 5, 6	133	F	57	O	4A, 4B, 5, 6
53	M	32	O	4A, 4B, 5, 6	134	M	62	AB	4A, 4B, 5, 6
54	F	30	O	4A, 4B, 5, 6	135	F	41	AB	1C
55	M	55	A	4A, 4B, 5, 6	135	F	41	AB	4A, 4B, 5, 6
56	F	48	O	4A, 4B, 5, 6	136	M	52	AB	4A, 4B, 5, 6
57	F	44	A	4A, 4B, 5, 6	137	M	46	AB	4A, 4B, 5, 6
58	M	42	B	2	138	F	36	AB	4A, 4B, 5, 6
59	F	43	A	4A, 4B, 5, 6	139	F	35	AB	4A, 4B, 5, 6
60	F	28	O	4A, 4B, 5, 6	140	M	58	AB	4A, 4B, 5, 6
61	F	29	A	4A, 4B, 5, 6	141	M	39	AB	4A, 4B, 5, 6
62	F	38	O	4A, 4B, 5, 6	142	M	46	AB	4A, 4B, 5, 6
63	F	49	O	4A, 4B, 5, 6	143	F	36	O	4A, 4B, 5, 6
64	F	29	O	4A, 4B, 5, 6	144	M	50	AB	4A, 4B, 5, 6
65	M	45	A	4A, 4B, 5, 6	144	M	50	AB	4A, 4B, 5, 6
66	F	43	A	4A, 4B, 5, 6	145	M	52	A	2D, 3A, 3B, 4C, 4D, 6
67	F	43	B	4A, 4B, 5, 6	146	M	30	AB	2D, 3A, 3B, 4C, 4D, 6
68	F	31	A	4A, 4B, 5, 6	147	M	48	A	2D, 3A, 3B, 4C, 4D, 6
69	F	28	A	4A, 4B, 5, 6	148	M	29	AB	2D, 3A, 3B, 4C, 4D, 6
70	M	27	O	4A, 4B, 5, 6	149	F	30	O	2D, 3A, 3B, 4C, 4D, 6
71	F	28	O	4A, 4B, 5, 6	150	F	33	AB	4C, 4D, 6
72	M	36	O	4A, 4B, 5, 6	151	M	28	O	4C, 4D, 6
73	F	26	O	4A, 4B, 5, 6	152	M	41	AB	4C, 4D, 6
74	F	24	A	4A, 4B, 5, 6	153	M	26	AB	4C, 4D, 6
75	F	57	O	4A, 4B, 5, 6	154	M	27	AB	4C, 4D, 6
76	M	30	O	4A, 4B, 5, 6	155	M	27	AB	4C, 4D, 6
77	M	20	O	4A, 4B, 5, 6	156	F	38	A	4C, 4D, 6
78	M	23	A	4A, 4B, 5, 6	157	F	45	AB	4C, 4D, 6
79	F	23	A	4A, 4B, 4C, 5, 6	158	F	65	O	2D, 3A, 3B, 4C, 4D, 6
80	F	55	O	4A, 4B, 4C, 5, 6	159	F	36	AB	4C, 4D, 6
81	M	31	A	4A, 4B, 4C, 5, 6	160	F	49	A	4C, 4D, 6
82	M	20	A	4A, 4B, 4C, 5, 6	161	F	43	A	4C, 4D, 6
83	F	26	O	4A, 4B, 4C, 5, 6	163	M	41	O	4C, 4D, 6
84	M	29	O	4A, 4B, 4C, 5, 6	164	M	37	AB	4C, 4D, 6
85	F	22	A	4A, 4B, 4C, 5, 6	165	F	25	O	4C, 4D, 6
86	M	26	A	4A, 4B, 4C, 5, 6	166	F	27	O	4C, 4D, 6
87	M	39	O	4A, 4B, 4C, 5, 6	167	M	47	O	2D, 3A, 3B, 4C, 4D, 6
88	M	52	A	4A, 4B, 4C, 5, 6	168	M	61	AB	4C, 4D, 6
					172	M	18	AB	2D, 3A, 3B

**Table 2:**
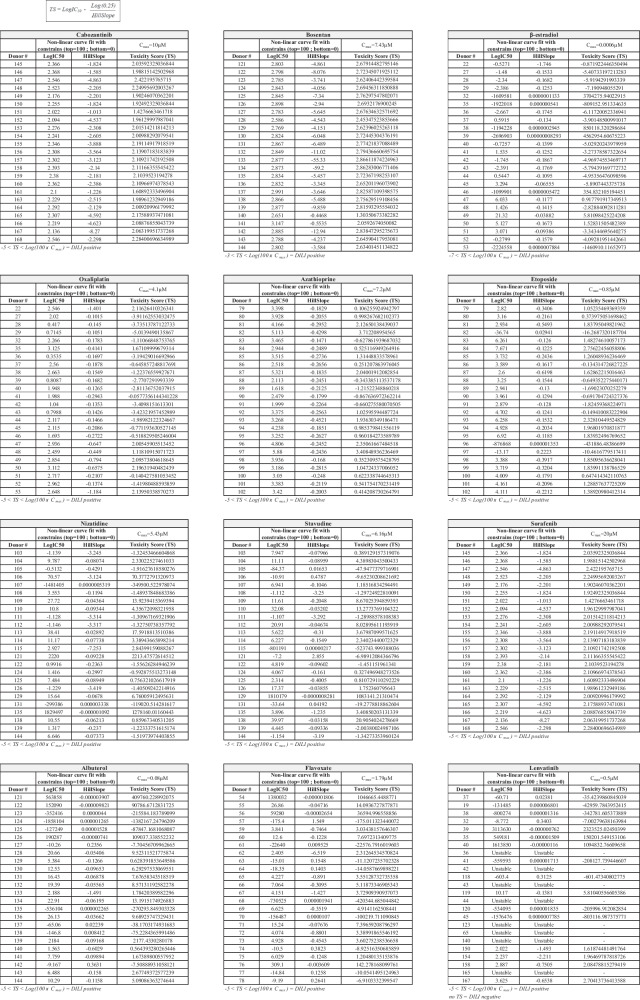
Toxicity score (TS)

**Table 3:** DILI prediction

Drugs	Clinically apparent liver injury	Number of DILI positive within a cohort of 24 individuals (%)	Predicted DILI risk	*C*_max_ reference
Albuterol	No	8.3	No	Proctor et al. 2017 [20]. Arch Toxicol
Flavoxate	No	4.2	No	Proctor et al. 2017 [20]. Arch Toxicol
β-Estradiol	Yes	54.2	Yes	Bircsak et al. 2021 [43]. Toxicology
Etoposide	Yes	75.0	Yes	Sipes et al. 2017 53]. Environ Sci Technol
Nizatidine	Yes	54.2	Yes	Sipes et al. 2017 [53]. Environ Sci Technol
Azathioprine	Yes	91.7	Yes	Proctor et al. 2017 [20]. Arch Toxicol
Oxaliplatin	Yes	100.0	Yes	Lurvink et al. 2021 [54]. Ann Surg Oncol
Bosentan	Yes	100.0	Yes	Proctor et al. 2017 [20]. Arch Toxicol
Stavudine	Yes	45.8	Yes	Proctor et al. 2017 [20]. Arch Toxicol
Lenvatinib	No	0.0	No	Ikeda et al. 2016 [55]. Clin Cancer Res
Cabozantinib	Yes	100.0	Yes	Jones et al. 2022 [56]. J Chromatogr Sci
Sorafenib	Yes	100.0	Yes	Brendel et al. 2011 [57]. Cancer Chemother Pharmacol

< 10% DILI-positive individuals in a cohort of 24 individuals = no DILI risk

Idiosyncratic DILI is generally difficult to predict and is usually not dose related contrary to intrinsic DILI that develops in a dose-dependent manner [22]. Interestingly, we found that azathioprine, a well-known iDILI drug [23], induces a reduction of about 35% of cell viability up to a concentration of 10× *C*_max_. This decrease of cell viability then remained unchanged even at higher doses of azathioprine while sorafenib displayed a clear dose-dependent reduction of cell viability (Fig. 4C). Our data suggest that donor-dependent educated spheroids might be capable of predicting iDILI risk.

Next, we assessed the reliability of our educated spheroid system to predict DILI risk. For that, we performed 2 independent experiments including 10 donors in the first cohort and 23 donors in the second cohort. Educated spheroids were treated with sorafenib, and we calculated the TS for each donor. As expected, we found that all donors from both cohorts were DILI positive upon exposure to sorafenib demonstrating that our results are consistent between 2 independent experiments (Fig. 4D).

**Fig. 5:**
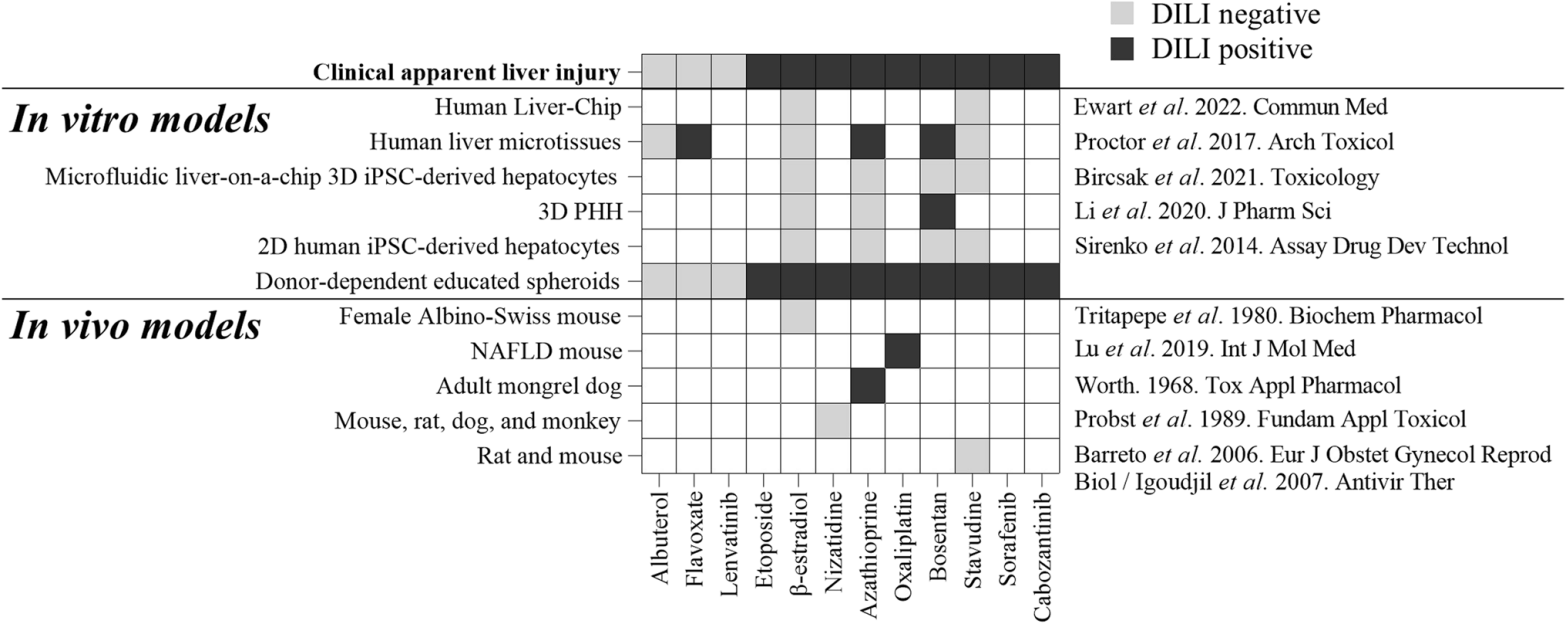
High predictive power of clinical apparent DILI risk of educated spheroids. Comparative analysis to current in vitro and animal models. Educated spheroids were generated using depleted serum from 109 donors. For each treatment group, educated spheroids from 24 donors were used (Table 1). A panel of drugs with or without clinical DILI concerns was used to test drug-induced hepatotoxicity. Heatmap shows overall predicted DILI risk for each drug. To compare the performance between educated spheroids and current preclinical models in predicting DILI risk, we extracted the data from the works cited on the right side of the heatmap

The performance of educated spheroids to predict DILI risk was assessed by comparing our results to those obtained from other in vitro and in vivo models. We found that educated spheroids correctly predicted clinical DILI in 9 drugs out of 9 and did not falsely mark albuterol, flavoxate, and lenvatinib as toxic, yielding a sensitivity and a specificity of 100% (Fig. 5). Meanwhile, other in vitro models and animal models were not capable to detect β-estradiol and stavudine-mediated DILI. Taken together, these data demonstrate that the educated spheroid system is more sensitive than current preclinical models to predict clinical DILI risk.

**Fig. 6:**
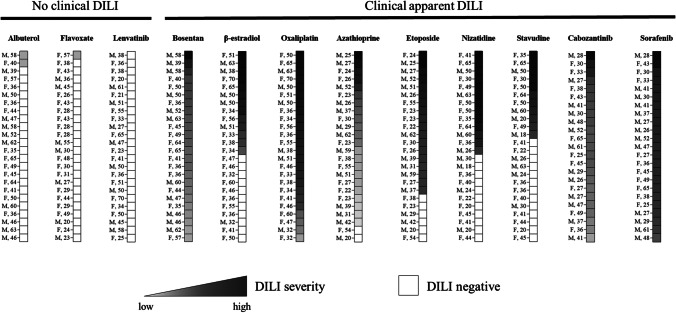
DILI risk stratification and severity grades. Data from a panel of 12 drugs (3 without clinical apparent liver injury and 9 with clinical apparent hepatoxicity) are reported as heatmaps. Each cell represents one donor. The sex and the age of the donor are reported on the left side of each cell. The degree of severity is determined by the TS (Table 2)

As non-genetic host factors that are associated to DILI development are age [24, 25] and sex [26–28], we analyzed age- and sex-associated DILI risk and DILI severity upon treatment with clinical DILI positive drugs. Figure 6 shows the risk for DILI development and the degree of severity (ranked accordingly to the TS) for each donor included in the study. As expected, cohorts treated with non-toxic drugs (flavoxate, albuterol, and lenvatinib) did not have more than 2 donors out of 24 (8.3%) who displayed a low DILI-positive risk while those treated with DILI-positive drugs have at least 11 donors (stavudine) out of 24 (45.8%) who showed a clear DILI-positive risk at different degrees of severity (Fig. 6). We then analyzed how much age and sex influence DILI risk and severity. We found that DILI risk is associated with the sex of the donor for β-estradiol (η_p_^2^ = 0.1595, *p* = 0.0532), while it is associated with the age of the donor for nizatidine (η_p_^2^ = 0.3414, *p* = 0.0027) (Fig. 6; Table 4). From 9 DILI-positive drugs tested, we found that the severity of DILI is associated with the age of the donors for β-estradiol (*R*^2^ = 0.3298, *p* = 0.0401) and oxaliplatin (*R*^2^ = 0.2247, *p* = 0.0193) (Fig. 6, Table 4). Overall, our data confirm that age and sex are host risk factors for DILI for some medications [29–31].

**Table 4:** Sex- and age-associated DILI risk

	Bosentan	β-Estradiol	Oxaliplatin	Azathioprine	Etoposide	Nizatidine	Stavudine	Cabozantinib	Sorafenib
Sex-associated DILI risk (ηp2)	-	0.1595* (*p* = 0.0532)	-	0.0004081 (*p* = 0.9253)	0.006442 (*p* = 0.7093)	0.1009 (*p* = 0.1303)	0.08606 (*p* = 0.1641)	-	-
Age-associated DILI risk (ηp2)	-	0.05065 (*p* = 0.2904)	-	0.001707 (*p* = 0.8480)	0.001493 (*p* = 0.8577)	0.3414* (*p* = 0.0027)	0.09124 (*p* = 0.1514)	-	-
Sex-associated DILI severity (ηp2)	0.08351 (*p* = 0.1708)	0.08956 (*p* = 0.3206)	0.02346 (*p* = 0.4749)	0.0002726 (*p* = 0.9419)	0.01233 (*p* = 0.6609)	0.1967 (*p* = 0.1291)	0.1824 (*p* = 0.1901)	0.003898 (*p* = 0.7772)	0.003037 (*p* = 0.8028)
Age-associated DILI severity (*R*^2^)	0.00926 (*p* = 0.6546)	0.3298* (*p* = 0.0401)	0.2247* (*p* = 0.0193)	0.04116 (*p* = 0.3652)	0.01185 (*p* = 0.7111)	0.1075 (*p* = 0.2741)	0.07678 (*p* = 0.4094)	0.06530 (*p* = 0.2392)	0.06480 (*p* = 0.2411)

According to Cohen’s guidelines ηp2 > 0.13 means large effect

10.4324/9780203771587

## Discussion

Drug-induced hepatotoxicity is a major challenge in drug development and personalized medicine [32]. Indeed, 90% of drugs that passed preclinical testing fail clinical trials because of liver toxicity [33]. Moreover, treatment discontinuation due to hepatotoxicity occurred in 20 to 40% of patients [34]. These observations suggest that an improvement of preclinical testing of DILI is urgently needed for the development of safer medications. We present here an easy to set up, to handle, and affordable model that reproduces the variability among people. This model makes possible the analysis of DILI risk in a population and thus de-risking failure when entering first-in-human trials. Furthermore, it also provides a way to give a more robust safety profile to a drug when it is used in an exploratory study in clinical trials phase 2.

One major drawback of currently available models to predict DILI risk is their inability to generate a functional donor-dependent liver specific microenvironment. Indeed, cholestasis is the main cause of DILI and is associated with an alteration of bile canaliculi functions [35]. As such, these canalicular structures are required for cholestasis toxicity detection [36]. We showed that educated spheroids trigger a spontaneous formation of bile canaliculi suggesting that our model is able to predict cholestasis toxicity (Fig. 1). Moreover, we demonstrated that the magnitude and the pattern of hepatic stellate cells activation is donor-dependent, and consequently, we observed a spontaneous donor-dependent deposition of ECM components (collagen and fibronectin) that are well known to influence DILI occurrence [37] (Fig. 1). In-depth analysis of the model revealed that important metabolic and signaling pathways were altered in educated spheroids (Fig. 2), including glycolysis and response to stimuli. Interestingly, we found a downregulation of genes that are associated with cancer in educated HepG2-based spheroids suggesting a trend towards normal primary human hepatocytes [38]. Finally, cell lines such as HepG2 or Huh7 are generally of limited use for predicting drug-induced hepatotoxicity because of the low expression of ADME genes as compared to the liver, making that they cannot detect drug toxicity mediated by metabolism [39]. We demonstrated that educating spheroids with donor’s depleted serum increased the basal CYP3A4 activity by 2 to 19 times (Fig. 2). Moreover, this activity was enhanced up to 80 times when educated spheroids were treated with a drug suggesting an upregulation of the drug metabolizing capacity of the cells (Fig. 3). This drug metabolizing capacity of educated spheroids was further confirmed by our results showing that educated spheroids can predict azathioprine- [40], nizatidine- [41], and etoposide- [42] mediated hepatoxicity, 3 compounds from which the mechanism of liver injury is primarily caused by their toxic metabolites (Fig. 4). Taken together, our data demonstrate that educating spheroids with donor’s depleted serum permits to obtain a functional donor-dependent liver specific microenvironment with an enhanced drug metabolizing capacity of HepG2 cells sufficiently to detect the hepatotoxicity induced by drug metabolites.

Primary human hepatocytes are generally used to study drug-induced liver injury. However, and despite they highly express AMDE genes, their capacity to predict DILI has shown limitations as they could not detect clinical DILI for some drugs such as stavudine or β-estradiol [20]. Attempts to use liver organoids derived from pluripotent stem cells to assess DILI were also unsatisfactory, although they retain the genetic background of the donor from who they derived from [43–45]. Indeed, liver cancer organoids are difficult to generate and with a poor success rate, while healthy liver organoids are typically arranged as monolayer of cells forming cysts making them imperfect models [46, 47]. Moreover, liver organoids require artificially predefined amount of Matrigel or synthetic ECM scaffolds, and thus, they do not reproduce the donor-dependent composition of ECM [48]. All these constrains make that current in vitro models have a limited capacity to predict DILI risk (Fig. 5). Animal models are also extensively used to analyze drug-induced hepatotoxicity. However, there are evidence that in vivo models are bad predictors of drug-induced toxicity in human [1] (Fig. 5). With a high sensitivity and specificity on the predictivity of clinical apparent DILI risk, educated spheroids appear to be a valuable option to analyze drug-induced liver injury easily and accurately, helping drug development pipelines.

Non-genetic factors contribute to the development of DILI too [49]. Indeed, elderly people are generally considered at high risk for DILI for some drugs [24], and an age cut-off point was estimated at 52 years old for high risk of adverse drug reactions [47]. Sex is considered as a non-genetic risk factor for DILI for some medications as well [49–52]. The good performance of educated spheroids in predicting DILI risk based on the age and the sex of the donor (Fig. 6) makes this model interesting to preclinically fine tune the safety profile of the people for whom the medication is dedicated.

Last but not least advantage of the educated spheroid model is its affordability as compared to current sophisticated in vitro models such as primary liver cells or organoids. Indeed, using educated spheroids to assess clinical DILI risk is barely more expensive than cell lines, and it is clearly financially much competitive than PHH or organoids.

## Conclusion

In summary, we describe here the first donor-dependent multicellular spheroid model that utilizes our patented cell education technology to assess, with a high specificity and sensitivity, the interindividual DILI risk. To our knowledge, this is a unique preclinical model that offers a way to analyze DILI risk based on non-genetic factors such as age or/and sex confirming therefore the safety of a drug before entering clinical trials. Thus, this new preclinical model will be of great interest for pharmaceutical companies that invest billions of dollars in drug development, reducing the cost and de-risking failures.

## Data Availability

The authors declare that the main data supporting the findings of this study are available on reasonable request with permission of PredictCan Biotechnologies SAS.
